# Consolidating the association of biallelic *MAPKAPK5* pathogenic variants with a distinct syndromic neurodevelopmental disorder

**DOI:** 10.1136/jmg-2022-108566

**Published:** 2022-12-29

**Authors:** Reza Maroofian, Stephanie Efthymiou, Mohnish Suri, Fatima Rahman, Maha S Zaki, Shazia Maqbool, Najwa Anwa, Victor L Ruiz-Pérez, Shira Yanovsky-Dagan, Orly Elpeleg, Sniya Sudhakar, Kshitij Mankad, Tamar Harel, Henry Houlden

**Affiliations:** 1 Department of Neuromuscular Disorders, UCL Queen Square Institute of Neurology, London, UK; 2 Clinical Genetics Service, Nottingham University Hospitals NHS Trust, Nottingham, UK; 3 Department of Developmental - Behavioral Pediatrics, University of Child Health Sciences & The Children’s Hospital, Lahore, Pakistan; 4 Clinical Genetics Department, Human Genetics and Genome Research Institute, National Research Centre, Cairo, Egypt; 5 Instituto de Investigaciones Biomédicas "Alberto Sols", Consejo Superior de Investigaciones Científicas (CSIC), Universidad Autónoma de Madrid (UAM), and CIBER de Enfermedades Raras (CIBERER), Madrid, Spain; 6 Faculty of Medicine, Hebrew University of Jerusalem, Jerusalem, Israel; 7 Department of Genetics, Hadassah Medical Center, Jerusalem, Israel; 8 Department of Radiology, Great Ormond Street Hospital for Children, London, UK

**Keywords:** Genetic Variation, High-Throughput Nucleotide Sequencing, Phenotype, Genotype

## Abstract

**Background:**

MAPK-activated protein kinase 5 (MAPKAPK5) is an essential enzyme for diverse cellular processes. Dysregulation of the pathways regulated by MAPKAPK enzymes can lead to the development of variable diseases. Recently, homozygous loss-of-function variants in *MAPKAPK5* were reported in four patients from three families presenting with a recognisable neurodevelopmental disorder, so-called ‘neurocardiofaciodigital’ syndrome.

**Objective and methods:**

In order to improve characterisation of the clinical features associated with biallelic *MAPKAPK5* variants, we employed a genotype-first approach combined with reverse deep-phenotyping of three affected individuals.

**Results:**

In the present study, we identified biallelic loss-of-function and missense *MAPKAPK5* variants in three unrelated individuals from consanguineous families. All affected individuals exhibited a syndromic neurodevelopmental disorder characterised by severe global developmental delay, intellectual disability, characteristic facial morphology, brachycephaly, digital anomalies, hair and nail defects and neuroradiological findings, including cerebellar hypoplasia and hypomyelination, as well as variable vision and hearing impairment. Additional features include failure to thrive, hypotonia, microcephaly and genitourinary anomalies without any reported congenital heart disease.

**Conclusion:**

In this study, we consolidate the causality of loss of MAPKAPK5 function and further delineate the molecular and phenotypic spectrum associated with this new ultra-rare neurodevelopmental syndrome.

## Introduction

*MAPKAPK5* encodes MAPK-activated protein kinase 5, also known as p38-regulated and-activated kinase (PRAK) or MK5, which is a serine/threonine protein kinase that was first characterised as a protein kinase similar to MAPKAPK2 (MK2). It is activated through a direct MAPK-dependent pathway to initiate and regulate diverse cellular processes, such as proliferation, differentiation, apoptosis and gene expression, in response to various external stimuli.^1^

Improper signalling or dysregulation of the cascades regulated by various MAPK enzymes can induce the development or progress of different diseases, such as cancers, diabetes or developmental disorders. A link between Alzheimer’s disease and reduced levels of MAPKAPK5 has been proposed.^2^ In addition, two recent studies reported four cases harbouring homozygous frameshift and nonsense *MAPKAPK5* variants with a Neurocardiofaciodigital syndrome (OMIM 619869). The patients presented with severe global developmental delay (GDD), facial dysmorphism, vision and hearing impairment, synpolydactyly, brain anomalies, and variable congenital cardiac and urological defects.^3^

In this study, we report three additional cases from three unrelated families with homozygous *MAPKAPK5* likely pathogenic*/*pathogenic variants. They presented similarly to the previously reported cases with a recognisable syndromic neurodevelopmental disorder (NDD) associated with severe GDD/intellectual disability (ID), distinct facial morphology and digital and brain abnormalities, as well as variable hearing and vision impairments without reported congenital cardiac defects.

## Materials and methods

### Human participants and clinical and genetic investigations

**Figure 1:**
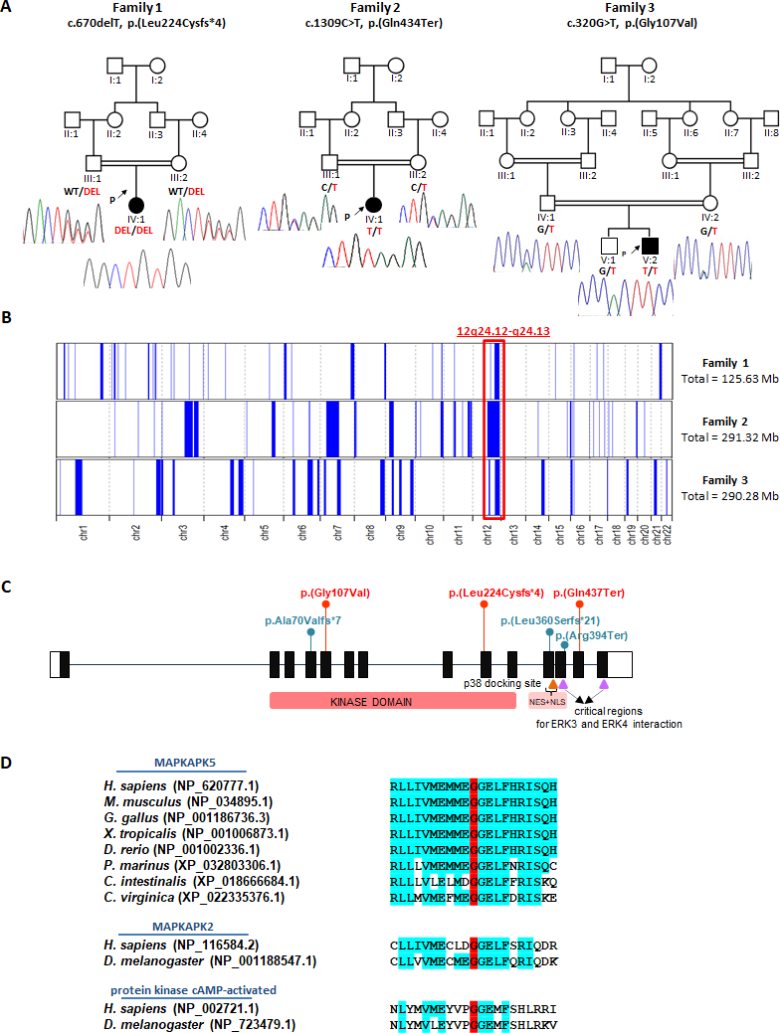
Identification of *MAPKAPK5* pathogenic variants in patients with a developmental disorder. (A) Family pedigrees of the three probands (arrows) with homozygous *MAPKAPK5* pathogenic variants described in this article. (B) Overview of the whole regions of homozygosity in the genome of each case. The block of homozygosity surrounding the MAPKAPK5 variants (indicated in red).^11^ (C) Schematic representation of *MAPKAPK5* exon structure showing the position of the variants. Exons are represented with filled boxes and empty boxes designate the UTR regions. The *MAPKAPK5* variants identified in this study are in red and previously reported pathogenic variants in blue. Functional domains of the MAPKAPK5 protein (MK5) including the N-terminal kinase domain, and signals for nuclear export (NES) and nuclear localisation (NLS) are depicted. The site for p38^MAPK^ docking, which overlaps with the NES+NLS domain, is indicated with an orange triangle. The purple triangles designate two regions (residues 383–393 and 460–465) which were demonstrated to be critical for MK5 interaction with both ERK3 and ERK4.^8^ (D) Partial amino acid sequence alignment demonstrating evolutionary conservation of Gly107 (highlighted in red) among MK5 protein homologues from different organisms. Conservation of this residue in the MK5 homologue protein MAPKAPK2 (MK2), and in the catalytic subunit of cAMP-activated protein kinase from human and *Drosophila melanogaster (D. melanogaster* (fruit fly)) is also shown. For the cAMP-activated protein kinase, the sequence of the catalytic subunit alpha (*Homo sapiens*) and the catalytic subunit 1 (*D. melanogaster*) are shown. Residues identical to human MK5 are highlighted in blue. Protein reference sequences used for the alignment are next to the name of each organism. Vertebrates: *Homo sapiens* (*H. sapiens*), *Mus musculus* (*M. musculus*), *Gallus gallus* (*G. gallus*), *Xenopus tropicalis (X. tropicalis* (tropical clawed frog)), *Danio rerio (D. rerio, zebrafish*), *Petromyzon marinus (P. marinus* (sea lamprey)). Invertebrates: *Ciona intestinalis (C. intestinalis* (vase tunicate)), *Crassostrea virginica* (*C. virginica* (easter oyster)).

We evaluated three individuals from three consanguineous south-Asian, middle-Eastern and north-African families (figure 1A). Detailed clinical features, as well as family history, brain MRI images and clinical photos, were obtained from three affected individuals and reviewed by a group of clinical geneticists (MS and MZ), paediatricians (FR and SM), a neurologist (HH) and two paediatric neuroradiologists (KM and SS), and compared with the data from previously reported families. Exome sequencing (ES) and Sanger segregation analysis were performed separately at two different laboratories as previously described.^4 5^

## Results

### Clinical and genetic characteristics

**Figure 2:**
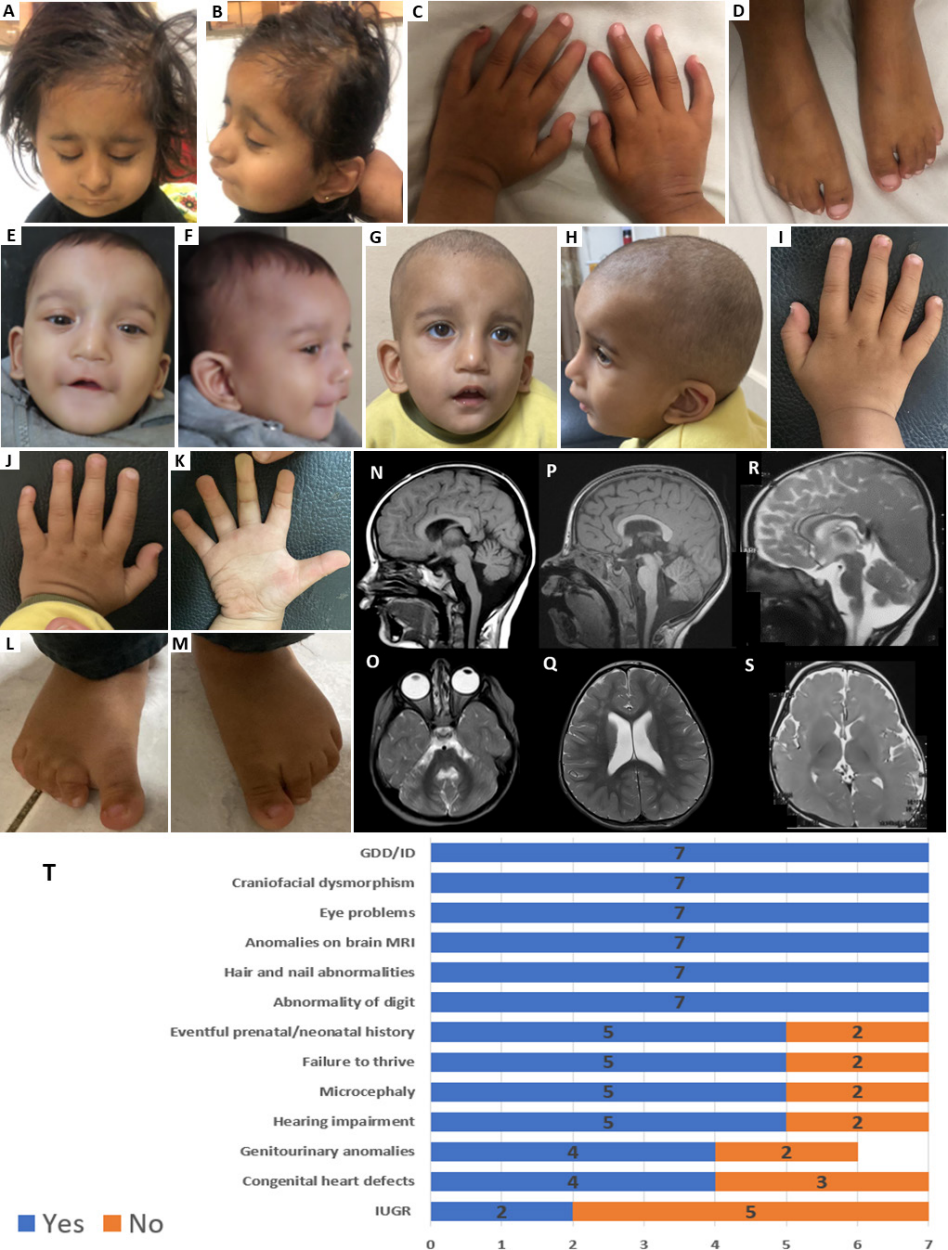
Frontal and lateral facial photographs of Proband 1 (A, B), photographs of hands (C) and feet (D), Facial photographs show brachycephaly, sparse scalp hair, bitemporal narrowing, straight eyebrows, triangular nose with full tip, hypoplastic nares, low-set columella, prominent medial maxillary incisors, full and tented upper lip, maxillary overbite and micro-retrognathia. Photographs of hands show short index fingers with absent nails and terminal phalanx. Photographs of feet show prominent sandal gap and relatively short fifth toes. Frontal and lateral photographs of Proband 3 in infancy (E, F) and at 1 year and 11 months (G, H). Photographs of hands (I–K) and both feet (L, M). Facial photographs show craniofacial asymmetry, depression over glabella, sparse eyebrows, shorter left palpebral fissure, hypertelorism, infraorbital creases, full nasal tip, short columella, low-set ears, absent superior crus of antihelix, uplifted ear lobes, small mouth, full tented upper lip, thin lower lip vermilion, maxillary overbite and retrognathia. Photographs of hands show mild syndactyly between the second and third fingers of his right hand, an incomplete single transverse palmar crease and normal fingers and nails. Photographs of feet show broad big toes, short second to fifth toes, and prominent sandal gap with deep groove over medial aspect of second toe. Case 1—N: Sagittal T1 weighted image showing superior cerebellar vermian and pontine atrophy, along with cerebellar vermian hypoplasia. The frontal lobe was noted to be relatively underdeveloped with anterior callosal hypoplasia. O: Axial T2 weighted image shows additional finding of cerebellar dysfoliation. Case 2—P: Sagittal T1 weighted image showing cerebellar vermian hypoplasia. Relative frontal lobar underdevelopment is noted along with hypoplasia of the anterior aspect of corpus callosum. Q: Axial T2 weighted image shows unspecific foci of white matter hyperintensities along with prominent lateral ventricles due to cerebral underdevelopment. Case 3—R: Sagittal T2 weighted image shows frontal atrophy associated with anterior callosal hypoplasia and mild cerebellar vermian hypoplasia. S: Axial T2 weighted image shows grossly delayed maturation of myelin. (T) Comparison of the main clinical characteristics of *MAPKAPK5*-related neurodevelopmental disorder among seven cases. Blue and orange colours indicate presence and absence of the features, respectively.

Proband 1 is a Pakistani girl in her early childhood, born to first-degree cousins with no family history of neurological diseases. Antenatal ultrasound scans noted decreased fetal movements and her mother had pregnancy-induced hypertension. She was born at term following an uneventful delivery; however, she was hypotonic and showed failure to thrive. She developed meningitis at the age of 2 years. She had severe GDD since early life; she was able to sit unsupported at the age of 18 months and started to walk with support by 30 months of age. Additionally, she had severe speech delay and could only say two to three words at the age of 4 years old. Her developmental delay was severe-to-profound with a Developmental Quotient (DQ) of 8 months at the age of 4 years, and she cannot perform basic activities. Her parents noticed that she started to develop wide-based gaits with balance problem at the age of 18 months. She is currently at level IV, according to Gross Motor Function Classification System (GMFCS). In addition to the severe developmental delay, she had facial dysmorphic features, including microbrachycephaly, sparse scalp hair, bitemporal narrowing, straight eyebrows, deep-set eyes, bilateral ptosis (right more than left), epicanthic folds, triangular nose with full tip, hypoplastic nares, low-set columella, prominent medial maxillary incisors, long philtrum, full and tented upper lip, maxillary overbite and micro-retrognathia (figure 2A and B). She had short index fingers with absent nails, slightly short fifth fingers with mild clinodactyly and camptodactyly, and a prominent sandal gap in her feet with relatively short fifth toes (figure 2C and D). Hearing assessment and audiometry indicated bilateral sensorineural hearing loss. Neurological findings included muscle weakness, hypotonia and diminished reflexes, broad-based gait, dyskinetic movements, abnormal hand movements and teeth grinding. She had feeding difficulties and was only able to eat semisolid food. General system examination did not find any abnormalities in the heart, gut, kidneys and lungs. Brain MRI at the age of 4 years revealed superior cerebellar vermian and pontine atrophy, along with cerebellar vermian hypoplasia and generalised cerebellar dysfoliation. The frontal lobe was noted to be relatively underdeveloped with anterior callosal hypoplasia (figure 2N and O).

Exome Sequencing of the proband uncovered a novel homozygous frameshift variant in *MAPKAPK5* (NM_139078.3:c.670del, p.Leu224CysfsTer4). The variant was located within an ~15 Mb region of homozygosity and both unaffected parents were carriers (figure 1A–C).

Proband 2 is a girl in her middle childhood, born to Arab first cousin parents. She had an unremarkable prenatal history and was born at 37 weeks’ gestation, at a birth weight of 1825 g (small for gestational age, Z-score −2.53) and head circumference of 28.5 cm (Z-score −3.07). After birth, she was pale and hypotonic and required intubation and life support. Her development was severely delayed; she started to walk at 30 months of age and had severe ID with DQ of 58 (age of 2 years). At the age of 7.8 years old, she can form words and sentences which are mainly incomprehensible. Facial dysmorphism included brachycephaly, highly arched eyebrows with medial flaring, narrow palpebral fissures, low columella, low-set ears and small chin (figure 2A). She had short fingers with mild clinodactyly and camptodactyly of the fifth fingers and bilateral minimal syndactyly of the second and third toes. Additional findings included astigmatism, bilateral optic disc colobomas, webbed neck and pectus excavatum. Neurological examination was normal apart from suspected hearing loss. General systemic examination with echocardiography and abdominal ultrasound were unremarkable. She had a history of bowel and bladder incontinence until the age of 6 years and 6 months. Brain MRI revealed cerebellar vermian hypoplasia and frontal lobar underdevelopment, along with hypoplasia of the anterior aspect of corpus callosum. -Non-specific foci of white matter hyperintensities were also shown in the frontal lobes (figure 2P and Q).

Trio ES detected a novel homozygous nonsense *MAPKAPK5* variant (NM_139078.3:c.1309C>T, p.Gln437Ter) residing within a 55.2 Mb block of homozygosity, with both unaffected parents being heterozygous for the variant (figure 1A–C).

Proband 3 is a toddler boy born to first-degree consanguineous Egyptian parents with no previous family history of neurological diseases. He had a prenatal history of oligohydramnios and was born at 39 weeks gestation. Following birth, he was kept in an incubator for 7 days due to tachypnoea. At birth, he exhibited hypotonia and genital anomalies, including small phallus, shawl scrotum and absent left testis. He showed mild developmental delay and excessive smiling and clapping of hands. On speech assessment at the age of ~2 years, he was able to pronounce only few words. He showed failure to thrive with weight of 10 kg (−2 SD), length of 83 cm (−1 SD) and head circumference of 45.5 cm (−2.3 SD). There was no history of seizures. Neurological examination was normal apart from hypotonia and mild inconsistent squint. Dysmorphic features include brachy-plagiocephaly with craniofacial asymmetry, depression over glabella, sparse eyebrows, bilateral ptosis, shorter left palpebral fissure, hypertelorism, infraorbital creases, full nasal tip, short columella, low-set ears, absent superior crus of antihelix, uplifted ear lobes, small mouth, full tented upper lip, thin lower lip vermilion, maxillary overbite and retrognathia (figure 2E–H). He had short fifth fingers with mild clinodactyly and camptodactyly, very mild syndactyly between the second and third fingers of his right hand, an incomplete single transverse palmar crease on both sides, normal fingers and nails, broad big toes, short second to fifth toes, and prominent sandal gap with deep groove over medial aspect of second toe (figure 2I–M). He had normal hearing, mild feeding difficulty and no other medical problems, except for recurrent vomiting in early life. Ultrasound of the abdomen and pelvis showed mild left tunical hydrocele and the left testis located in the left inguinal canal. The EEG was normal. Brain MRI taken at the age of 8 months old revealed gross immaturity of myelination (delayed or hypo-myelination pattern), along with mild cerebellar vermian hypoplasia and reduced parenchymal volume in the frontal lobes (figure 2R and S).

Exome sequencing of the proband found an ultra-rare homozygous missense variant in *MAPKAPK5* (NM_139078.3:c.320G>T, p.Gly107Val) which is within an ~20.5 Mb region of homozygosity, and both parents, as well as the unaffected brother, were heterozygous for the variant. Only three carriers were observed in around 1 600 000 alleles across multiple variant frequency databases (online supplemental table 1). It occurs at a highly evolutionary conserved amino acid residue with the protein kinase domain of MAPKAPK5 (CADD Score 27 and GERP score 5.01) and is unanimously predicated to be damaging, deleterious and pathogenic by employed in-silico prediction tools (figure 1A–D). The variant is classified as likely pathogenic according to the American College of Medical Genetics and Genomics and the Association for Molecular Pathology standards and guideline.

10.1136/jmg-2022-108566.supp2 Supplementary data

Online supplemental table 1 summarises detailed clinical features of all three patients and compares them to the four previously reported patients. The variants reported in this report have been deposited into the Leiden Open Variation Database (LOVD) at https://databases.lovd.nl/shared/genes/MAPKAPK5.

## Discussion

In this study, we present three new individuals (two females and one male with an age range of 2–7 years) affected by a syndromic NDD associated with homozygous variants in *MAPKAPK5*. Clinical features shared by all three of them include GDD/ID, brachycephaly, facial dysmorphism, digital anomalies, neuroradiological abnormalities and ocular defects. Other findings seen in some of the affected individuals included intrauterine growth restriction (IUGR), failure to thrive, microcephaly, hypotonia and hearing loss.

Horn *et al* 6 (2021) and Vecchio *et al* 3 reported four cases from three unrelated nuclear families who were homozygous for three different loss-of-function *MAPKAPK5* variants. The reported cases have very similar clinical findings and share phenotypic features with the three cases in this study, although with some variability. The most common features of the syndrome which have been found thus far in all seven cases (four females and three males aged between 19 months and 9 years) include GDD/ID, anomalies of the digits, characteristic craniofacial morphology, ocular anomalies of variable severity and brain malformations. Prenatal and neonatal history was remarkable in most cases (5/7), and failure to thrive (5/7), microcephaly (5/7), hearing impairment (5/7), congenital urogenital anomalies (5/6 but 3/3 males) and congenital heart diseases (4/7) were observed in the majority of the affected individuals. None of the patients were reported to have history of seizures at their last medical examinations. The key clinical findings and their occurrence in all seven patients are demonstrated in figure 2T. The three cases reported here have no congenital cardiac anomalies and have more variable digital anomalies with none having synpolydactyly seen in three of the four previously reported cases. The cardiac anomalies seen in this condition are variable, ranging from simple to complex.

Facial dysmorphic features were seen in all affected cases. The most frequently observed findings included sparse scalp hair and eyebrows, narrow palpebral fissures, full overhanging nasal tip and micro-retrognathia. Two of the previously reported cases had features reminiscent of Pierre Robin sequence with micro-retrognathia, glossoptosis and high-arched palate rather than the classical U-shaped or V-shaped posterior cleft palate. Digital anomalies are also a key diagnostic feature of this condition and can include short fingers, fingernail or toenail hypoplasia or dysplasia, broad big toes and synpolydactyly with an additional hypoplastic digit between the fourth and fifth toes in two cases. All affected cases had neuroradiological abnormalities. Findings seen most frequently included anterior hypoplasia of the corpus callosum with relative frontal cerebral underdevelopment and cerebellar vermian hypoplasia.

All three male patients had genitourinary anomalies. The genital anomalies were variable and included one or more of shawl scrotum, peno-scrotal transposition, hypospadias, unilateral hemiscrotal hypoplasia and anchored phallus. One female child had hydronephrosis with vesicoureteral reflux and one male child had unilateral renal hypoplasia.

There is considerable overlap between the phenotype of MAPKAPK5-related NDD and the syndrome of dysmorphic facies, renal agenesis, ambiguous genitalia, microcephaly, polydactyly and lissencephaly (DREAM-PL), which was reported in 2016 by Shaheen and colleagues.^7^ This entity is listed in OMIM as microcephaly, facial dysmorphism, renal agenesis and ambiguous genitalia (MFRG; OMIM 618142). This is an ultra-rare autosomal recessive disorder due to homozygosity for a recurrent missense variant, c.873G>A, p.(Thr247AlaFsTer21), in *CTU2*. Distinguishing features include a more severe presentation with early lethality, lissencephaly, severe microcephaly, overlapping fingers, post-axial polydactyly of the feet, talipes, agenesis of the corpus callosum and unilateral renal agenesis in DREAM-PL, and the unusual synpolydactyly, short fingers and fingernail and toenail dysplasia in MAPKAPK5-related NDD. Congenital heart disease can be seen in both conditions and in some instances, genetic testing will be required to distinguish between these disorders. There is also some overlap between the phenotype of MAPKAPK5-related NDD and Coffin-Siris syndrome, but the nail dysplasia in the former does not typically affect the nails of the fifth fingers or little toes. In addition, patients with the latter are coarser with sparse scalp hair but hypertrichosis over the body.

Considering the six variants identified thus far, MAPKAPK5-related NDD results from loss-of function of the MAPKAPK5 protein. Five variants lead to premature stop codons which should trigger nonsense-mediated mRNA decay (NMD) or delete essential MAPKAPK5 domains. Horn *et al* demonstrated reduced levels of *MAPKAPK5* mRNA and protein in fibroblasts from an individual homozygous for the p.(Ala70Valfs*7) variant. Gln437Ter is predicted to escape NMD, but the predicted protein, in the case of being stable, would lack critical C-terminal residues for MAPKAPK5 interaction with ERK3/4.^8 9^ For the Gly107Val variant, experiments need to be performed to determine how exactly this variant could possibly affect the structure and function of the protein. However, Gly107 is a highly conserved residue among MAPKAPK5 protein homologues from distantly related species and is located within the flexible hinge region connecting the N-terminal and C-terminal lobes of the kinase domain. Notably, Gly107 together with Gly108 form a Gly-Gly sequence which is present in several different protein kinases, including MK2 and c-AMP dependent protein kinase among others^10^ (figure 1D and online supplemental figure 1). Additionally, the alteration of Gly107Val is extremely rare in human populations and the clinical phenotype of the patient, in whom ES did not identify any other variants explaining the disorder, is strikingly similar to six other affected children with homozygous loss-of-function variants in *MAPKAPK5*. These findings strongly support the significance of the Gly107 residue for proper function of the protein.

10.1136/jmg-2022-108566.supp1 Supplementary data

In conclusion, this study reports three new patients with MAPKAPK5-related NDD, consolidating the gene-disease association, expanding the clinical and molecular spectrum of the disorder and further delineating this new ultra-rare, but recognisable syndrome.
