# Supplementary figures and images for: Consolidating the association of biallelic MAPKAPK5 pathogenic variants with a distinct syndromic neurodevelopmental disorder
Source: J Med Genet. 2022 Dec 29;60(8):791–6. doi: 10.1136/jmg-2022-108566 (PMC10423509; doi:10.1136/jmg-2022-108566)

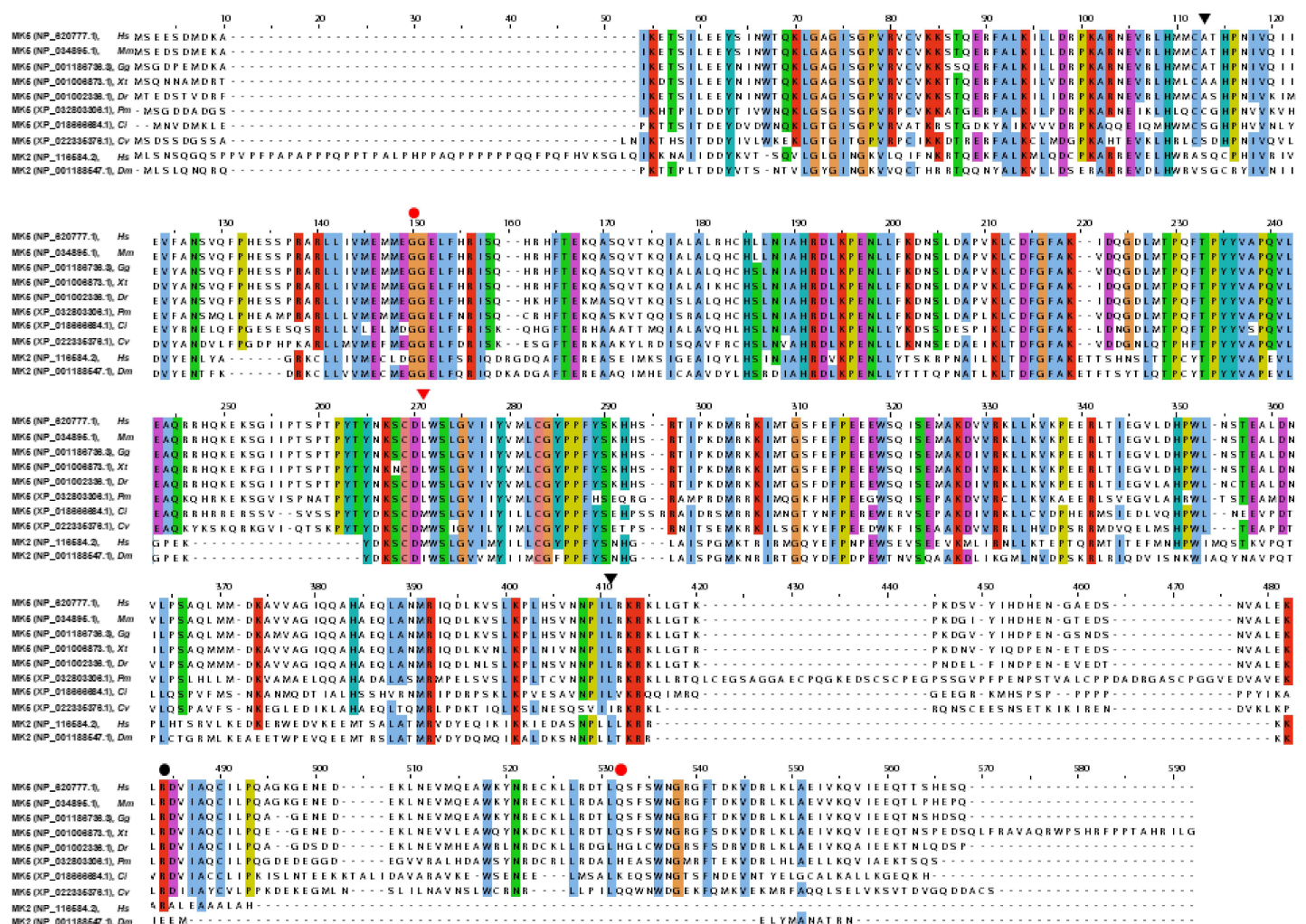

Supplement: Supplementary data [file jmg-2022-108566supp001.pdf]
